# Long-Term Outcome of Lower Extremity Bypass Surgery in Diabetic and Non-Diabetic Patients with Critical Limb-Threatening Ischaemia in Germany

**DOI:** 10.3390/biomedicines12010038

**Published:** 2023-12-22

**Authors:** Johanna Surmann, Philipp Meyer, Jasmin Epple, Thomas Schmitz-Rixen, Dittmar Böckler, Reinhart T. Grundmann

**Affiliations:** 1 Department of Vascular and Endovascular Surgery, University Hospital Heidelberg, 69120 Heidelberg, Germany; johanna.surmann@gmail.com (J.S.); philipp.mey@googlemail.com (P.M.); dittmar.boeckler@med.uni-heidelberg.de (D.B.); 2 Department of Vascular and Endovascular Surgery, University Hospital Frankfurt am Main, 60596 Frankfurt am Main, Germany; jas.epple@web.de; 3 Department of Vascular and Endovascular Surgery, Goethe University Frankfurt, 60629 Frankfurt am Main, Germany; schmitz-rixen@dgch.de; 4 German Institute for Vascular Healthcare Research (DIGG), German Society for Vascular Surgery and Vascular Medicine, 10115 Berlin, Germany

**Keywords:** bypass surgery, open revascularisation, diabetes, critical limb-threatening ischaemia, peripheral artery disease, long-term survival, major amputation

## Abstract

Aim: To present the short- and long-term outcomes of lower extremity bypass (LEB) surgery in patients with critical limb-threatening ischaemia (CLTI), comparing diabetic (DM) and non-diabetic (non-DM) patients. Methods: Retrospective analysis of anonymised data from a nationwide health insurance company (AOK). Data from 22,633 patients (DM: *n* = 7266; non-DM: *n* = 15,367; men: *n* = 14,523; women: *n* = 8110; mean patient age: 72.5 years), who underwent LEB from 2010 to 2015, were analysed. The cut-off date for follow-up was December 31, 2018 (mean follow-up period: 55 months). Results: Perioperative mortality was 10.0% for DM and 8.2% for non-DM (*p* < 0.001). Patients with crural/pedal bypasses (*n* = 8558) had a significantly higher perioperative mortality (10.3%) than those with above-the-knee (*n* = 7246; 5.8%; *p* < 0.001) and below-the-knee bypasses (*n* = 6829; 8.9%; *p* = 0.003). The 9-year survival rates in DM patients were significantly worse, at 21.5%, compared to non-DM, at 31.1% (*p* < 0.001). This applied to both PAD stage III (DM: 34.4%; non-DM: 45.7%; *p* < 0.001) and PAD stage IV (DM: 18.5%; non-DM: 25.0%; *p* < 0.001). Patients with crural/pedal bypasses had a significantly inferior survival rate (25.5%) compared to those with below-the-knee (27.7%; *p* < 0.001) and above-the-knee bypasses (31.7%; *p* < 0.001). Conclusion: Perioperative and long-term outcomes regarding survival and major amputation rate for CLTI patients undergoing LEB are consistently worse for DM patients compared to non-DM patients.

## 1. Introduction

The aim of this study was to analyse outcomes following lower extremity bypass (LEB) surgery in patients with critical limb-threatening ischaemia (CLTI), comparing patients with diabetes mellitus (DM) and patients without diabetes mellitus (non-DM). There are only a few major studies that have evaluated the long-term outcome of DM and non-DM patients following LEB surgery. Wallaert et al. [1] reported on 1977 LEBs from the Vascular Study Group of New England and found an increased rate of postoperative complications in DM compared to non-DM; long-term outcomes were not presented. Darling et al. [2] reported on 646 LEBs for CLTI and found no association between DM and perioperative complications or death. Nevertheless, the wound healing rate after 6 months was significantly worse in patients with insulin-dependent DM. Hicks et al. [3] observed no significant differences in 1-year primary patency, major amputation, or mortality following LEB and peripheral vascular intervention in DM and non-DM in a total of 2566 patients (including 500 patients with LEB). Hertzer et al. [4] reported on 650 LEBs performed by a single surgeon, with 85% performed for CLTI, and 48% of the patients being diabetics. DM was the only factor among several that influenced patient survival and limb preservation and did not have a measurable effect on graft patency. Finally, Lilja et al. [5] found no differences in mortality, cardiovascular death or major amputations between DM and non-DM in 1537 urgently planned open revascularisations for CLTI, including 569 DM. However, DM had significantly higher rates of stroke and myocardial infarction. 

All studies have the following in common: a relatively small number of cases, limited data on long-term outcomes, and inadequate differentiation between CLTI patients with and without tissue loss. Furthermore, the outcome depending on bypass level was not reported. As the cited studies show, the results are contradictory as to whether survival and amputation rates are worse in diabetics than in non-diabetics.

Therefore, the present study aims to determine, for the first time, short- and long-term outcomes after LEB in DM and non-DM with CLTI, differentiated by Fontaine stages III (rest pain) and IV (ulcer/gangrene) and bypass level (above-the-knee, below-the-knee, and crural/pedal), using a large from of a German health insurance company.

## 2. Materials and Methods

### 2.1. Study Design

In the present retrospective study, anonymised data from the nationwide AOK health insurance in Germany were analysed. The data were provided by the WIDO of “AOK—Die Gesundheitskasse”. Diagnoses and procedures were provided in form of ICD-10 codes (International Statistical Classification of Diseases and Related Health Problems, version 10) and OPS codes (Operation and Procedure Codes).

### 2.2. Patients

The study included all patients (*n* = 22,633) who underwent LEB between 2010 and 2015 (OPS codes: bypass level above-the-knee: 5-393.53; below-the-knee: 5-393.54; crural/pedal: 5-393.55, 5-393.56, 5-393.61, 5-393.62) with concomitant PAD Fontaine stages III or IV (ICD codes: 2010–2014: stage III: I70.22, stage IV: I70.23 and I70.24; 2015–2018: stage III: I70.23, stage IV: I70.24 and I70.25). Patients were grouped into those without (*n* = 15,367) and those with (*n* = 7266) a known diagnosis of type 2 diabetes mellitus (ICD code: E11.-) and compared for general patient characteristics, comorbidities, and perioperative complications as well as long-term survival and major amputation rates. Since only the month of death was known for deceased patients, all patients who died in the month of bypass surgery or the following month were documented as having died perioperatively. Follow-up ended for all patients on 31 December 2018. The mean follow-up period was 55 months (SD: 31.0 months).

### 2.3. Statistics

Data analysis was carried out using SPSS 28 (IBM Deutschland GmbH, Ehningen, Germany). To test for differences between each group, the chi-square test was used for non-metric variables. For metric variables, the Mann–Whitney U test was used. Kaplan–Meier estimates were used to analyse survival and major amputation rates during the 9-year follow-up. Differences between the curves were tested using the log-rank test. To assess the influence of patient characteristics and comorbidities on long-term survival, a univariate Cox regression analysis was performed. All analysed patient characteristics and comorbidities were included in the univariate analysis. All statistically significant variables from the univariate analysis were included in a multivariate analysis. *p*-values less than 0.05 were defined as statistically significant.

## 3. Results

### 3.1. Patients

Patient characteristics and comorbidities, differentiated by DM (*n* = 7266) and non-DM (*n* = 15,367), are shown in Table 1. The proportion of men in non-DM was 64.6%, compared to 63.2% among DM (*p* = 0.040). Non-DM had a mean age of 72.2 ± 10.8 years whereas the mean age of DM was 73.2 ± 9.7 years (*p* < 0.001). In both non-DM and DM, women were significantly older than men (non-DM: 76.8 ± 10.3 vs. 69.6 ± 10.3 years, *p* < 0.001; DM: 76.5 ± 9.4 vs. 71.3 ± 9.4 years, *p* < 0.001). PAD stage III was present in 30.2% of non-DM but in only 19.5% of DM (*p* < 0.001). Conversely, 69.8% of non-DM but 80.5% of DM had PAD stage IV (*p* < 0.001). Table 1 demonstrates significantly higher rates of comorbidity in DM compared to non-DM. Furthermore, this table lists the bypass level. For PAD stage IV, above- and below-the-knee bypasses were performed more frequently in non-DM compared to DM, while crural/pedal bypasses were more common in DM (43.7% vs. 39.2%; *p* < 0.001). For stage III, there was no difference between DM and non-DM in bypass level.

### 3.2. Perioperative Outcome

Table 2 shows the perioperative outcome. Overall, perioperative mortality was significantly lower in patients with PAD stage III compared to stage IV (4.5% vs. 10.3%; *p* < 0.001). A significantly higher perioperative mortality was observed in DM compared to non-DM (10.0% vs. 8.2%; *p* < 0.001). This was mainly driven by the higher perioperative mortality in PAD stage IV (11.2% vs. 9.9%; *p* = 0.009). In both DM and non-DM, the perioperative mortality of women was higher than that of men (non-DM 9.7% vs. 7.3%; *p* < 0.001; DM: 11.6% vs. 9.1%; *p* < 0.001). Table 2 presents the higher perioperative complication rate in DM compared to non-DM, with 56.4% of procedures being complication-free in DM as opposed to 69.2% in non-DM (*p* < 0.001). Major amputations occurred in 7.4% of DM vs. 5.8% of non-DM (*p* < 0.001), and minor amputations in 28.9% of DM vs. 17.4% of non-DM (*p* < 0.001).

Perioperative mortality was dependent on bypass level. Overall (DM and non-DM combined), patients with crural/pedal bypasses (*n* = 8558) had a significantly higher perioperative mortality of 10.3% compared to 8.9% (*p* = 0.003) in patients with below-the-knee bypasses (*n* = 6829) and 6.8% (*p* < 0.001) in patients with above-the-knee bypasses (*n* = 7246). The same applied to the major amputation rate, which was 10.2% for patients with crural/pedal bypasses, as opposed to 4.7% for patients with below-the-knee bypasses (*p* < 0.001) and 3.2% for patients with above-the-knee bypasses (*p* < 0.001).

### 3.3. Long-Term Survival in PAD Stages III and IV

**Figure 1:**
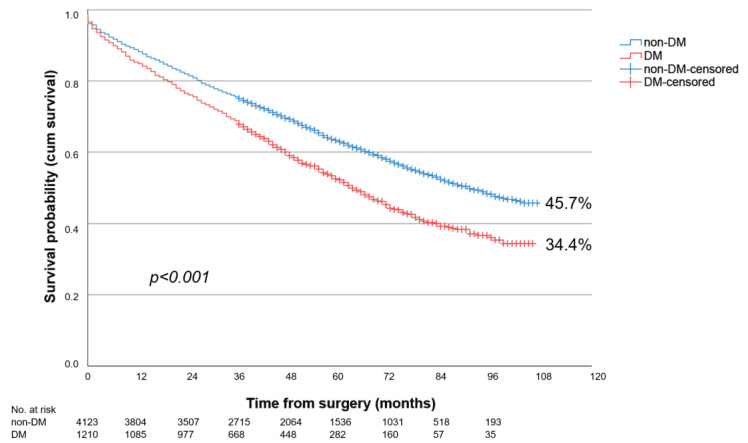
Nine-year survival of patients with diabetes (DM) or without diabetes (non-DM) and PAD Fontaine stage III following LEB.

**Figure 2:**
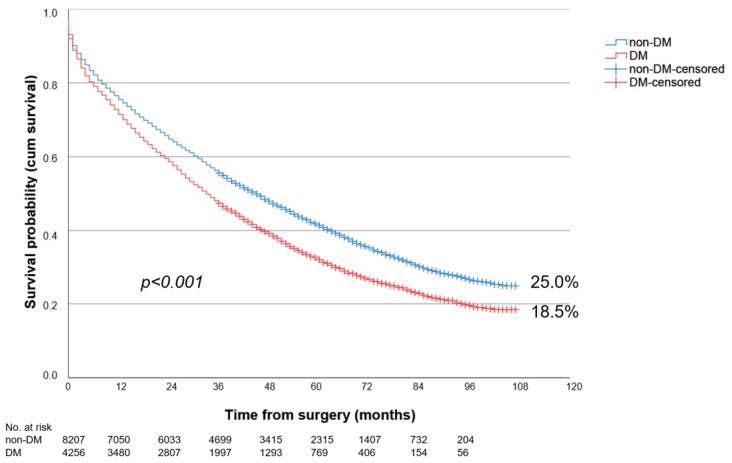
Nine-year survival of patients with diabetes (DM) or without diabetes (non-DM) and PAD Fontaine stage IV following LEB.

Overall, patients with PAD stage III showed the significantly better long-term survival over 9-year follow-up compared to patients with PAD stage IV (43.2% vs. 22.7%; *p* < 0.001). The long-term survival of DM patients was significantly worse, at 21.5%, compared to non-DM, at 31.1% (*p* < 0.001). This was true for both PAD stage III (DM: 34.4% vs. non-DM: 45.7%; *p* < 0.001) (Figure 1) and PAD stage IV (DM: 18.5% vs. non-DM: 25.0%; *p* < 0.001) (Figure 2).

### 3.4. Long-Term Survival Depending on Bypass Level

Patients with crural/pedal bypasses had significantly less favourable 9-year survival rates (25.5%) compared to patients with below-the-knee (27.7%; *p* < 0.001) and above-the-knee bypasses (31.7%; *p* < 0.001). For above- and below-the-knee bypasses, as well as for crural/pedal bypasses, the survival of DM was always worse compared to non-DM (Table 3). The primary influencing factors on long-term survival were PAD stage IV (HR: 1.62, CI: 1.55–1.69, *p* < 0.001) and renal insufficiency stages 3–5 (HR: 1.44, CI: 1.38–1.51, *p* < 0.001). Pre-existing DM also had a significant negative impact (HR: 1.10, CI: 1.05–1.14, *p* < 0.001) on long-term survival. Other factors influencing long-term survival are shown in Table 4.

### 3.5. Major Amputation Rates during Follow-Up in PAD Stages III and IV

Overall, the 9-year major amputation rate (ipsilateral and contralateral combined) in PAD stage IV was significantly higher than in PAD stage III (45.6% vs. 30.5%; *p* < 0.001). Furthermore, the major amputation rate was significantly higher (*p* < 0.001) in DM (45.3%) compared to non-DM (39.0%). This applied to both PAD stages III and IV (Table 5).

### 3.6. Major Amputation Rates during Follow-Up Depending on Bypass Level

Patients with crural/pedal bypasses had a significantly higher 9-year major amputation rate (50.5%) at follow-up compared to patients with above-the-knee bypasses (31.9%; *p* < 0.001) and below-the-knee bypasses (39.2%; *p* < 0.001). Among PAD stage IV patients, the major amputation rate for each bypass level was always significantly higher in DM compared to non-DM; in PAD stage III, this was only true for crural/pedal bypasses (Table 5).

## 4. Discussion

The present study investigated the outcome of LEB in DM and non-DM patients with PAD stage III and IV. In a large patient cohort, notably inferior outcomes were seen in DM compared to their non-DM counterparts. This disparity was evident in higher perioperative mortality, diminished long-term survival and increased long-term major amputation rates in DM. Furthermore, the outcome varied based on the PAD stage and the level of LEB. Patients in PAD stage III demonstrated significantly better outcomes compared to those in stage IV. Furthermore, individuals undergoing crural/pedal bypasses experienced markedly higher perioperative mortality and poorer long-term survival rates compared to those undergoing above- and below-the-knee bypasses. 

Short- and long-term outcomes after LEB in patients with CLTI and DM vs. non-DM have been reported, to a limited extent, with much smaller patient populations and shorter follow-up periods. Hicks et al. (2016) [3] compared outcomes following LEB in 355 DM vs. 145 non-DM. They found a 1-year mortality of 10% (DM) vs. 6% (non-DM) and a major amputation rate of 15% (DM) vs. 12% (non-DM), which were not significantly different. However, they did not distinguish between patients with rest pain and tissue loss, respectively, nor did they provide information on the outcome depending on bypass level. In the present study, a 1-year mortality of 24.8% vs. 19.8% (*p* < 0.001) was observed for DM vs. non-DM, with major amputation rates of 12.4% vs. 9.7% (*p* = 0.003) in PAD stage III and 23.0% vs. 19.5% in stage IV (*p* < 0.001).

Darling et al. [2] reported a perioperative mortality of 3.0% in insulin-dependent DM (*n* = 703), 1.5% in non-insulin-dependent DM, and 4.9% in non-DM (*n* = 329) after infrainguinal bypass grafting (BPG) or percutaneous transluminal angioplasty with or without stenting for CLTI. The results did not differ between open or endovascular revascularisation. These authors found 3-year survivals of 65% in noninsulin-dependent DM, 56% in insulin-dependent DM, and 51% in non-DM. The message of their study was that DM and non-DM did not differ significantly in perioperative mortality or long-term survival. In our patient cohort, the best 3-year survival was observed in non-DM with PAD stage III (75.6%) and the least favourable in DM with PAD stage IV (48.0%).

In a small propensity score-matched cohort of only 113 consecutive LEB, the 3-year survival rate was 57.6% in DM and 88.6% in non-DM (*p* = 0.01) [6]. However, no difference was found in patency (58.3% vs. 56%) and limb salvage rate (74.1% vs. 60.8%). These authors concluded that diabetes did not impact the bypass patency rate, but did negatively affect patient survival.

A similar conclusion had been drawn previously by AhChong et al. [7]. They analysed the outcomes of LEB for CLTI in 176 DM and 89 non-DM. Hospital mortality was significantly higher in DM (8% vs. 1%; *p* = 0.04) and patient survival at 5 years was significantly inferior in DM as compared to non-DM (33% vs. 43%; *p* = 0.03), but limb salvage rates were comparable. Like the other cited authors, AhChong et al. [7] considered neither bypass level nor PAD stage in their analysis. In the present study, diabetes had a negative impact on both patient survival and major amputation rates. The 5-year survival for non-DM was 54.3% as compared to 43.0% for DM (*p* < 0.001). After 5 years, major amputation rates of 37.8% (DM) vs. 31.1% (non-DM) were observed (*p* < 0.001).

Another single-centre study was described by Ballotta et al. [8]. They reported a total of 1407 LEB, 705 (50.2%) in 643 DM and 702 in 667 non-DM. Autogenous vein conduits were used in 87% of cases. Of the patients, 25.5% experienced rest, 38.0% experienced non-healing ulcer, and 36.6% experienced gangrene. There were no perioperative deaths observed. DM had significantly more major complications (16.7% vs. 11.8%; *p* = 0.02) as compared to non-DM. The 5- and 10-year survival rates were 51% and 34% in the DM group and 57% and 38% in the non-DM group. Major amputation rates did not differ over the long-term, with 8.4% in DM vs. 7.5% in non-DM. In conclusion, equivalent results were obtained in DM and non-DM. The extent to which the results of a single specialised centre can be generalised may be questioned. It is more likely that a selection bias exists, especially because the presentation of the results did not strictly differentiate between CLTI patients with rest pain (Fontaine stage III) and ulcer/gangrene (stage IV).

In a retrospective analysis of the Vascular Quality Initiative infrainguinal bypass module, McGinigle et al. [9] included 17,517 nondiabetic and 5194 patients with low-severity/controlled diabetes and 8102 patients with poorly controlled diabetes; in this cohort, approximately 20% of the patients with uncontrolled diabetes underwent bypass surgery for claudication. Those with haemoglobin A1c (HbA1c) of > 10% had an 81% increase in major adverse cardiac events and 31% increase in major adverse limb events (MALEs) within 30 days of revascularisation compared to patients without diabetes. The degree of uncontrolled diabetes was directly associated with significantly poorer 30-day surgical outcomes, including limb loss. Long-term data were not presented. In another retrospective analysis of registry data from the Vascular Quality Initiative of 7727 patients with LEB, poor preoperative glycaemic control in diabetic individuals, particularly in those without critical limb ischaemia, was associated with an increased risk of in-hospital limb events [10]. 

Elbadawi et al. [11] found, from the National Inpatient Sample (NIS) database, a final cohort of 1,222,324 hospitalisations with a primary diagnosis of CLTI and a secondary diagnosis of DM. Of those, revascularisation was performed in 410,829 (28%). After propensity matching, those undergoing surgical revascularisation had higher rates of in-hospital mortality (1.9% vs. 1.6%) and lower rates of major amputation (5.4% vs. 7.0%) as compared to those with endovascular revascularisation. They observed an increasing number of hospitalisations for CLTI among patients with DM over the study period. The PAD stage (ischaemia or tissue loss), the type of surgical revascularisation, a comparison of diabetic and non-diabetic patients, and long-term outcome data were not presented. 

De Donato et al. [12] presented a prospective multicentre cohort study with 12-month follow-up enrolling patients (*n* = 287) with CLTI undergoing open, endovascular, or hybrid lower extremity revascularisation. In this study, Rutherford class (≥5) and below-the-knee disease were significant predictors of amputation. In the stepwise multivariate analysis, diabetes could not be identified as a risk factor (*p* = 0.15). Outcome data with respect to the type of revascularisation were not given and a comparison between diabetics and non-diabetics was not performed. 

Fernando et al. [13] performed a systematic literature search to find studies using a validated measure of frailty in individuals with CLTI and/or DFUs. Ten cohort studies were included. The prevalence of frailty in people presenting with CLTI ranged from 27% to 88% and was 71% in people with DFUs. They concluded that the presence of frailty in both people with CLTI and DFUs is likely associated with substantially higher complexity at presentation followed by a greater risk for readmission, amputation, and death during follow-up. A comparison of the outcomes in diabetics vs. non-diabetics was not given and surgical revascularisation was not analysed.

Malyar et al. [14] analysed data from a German health insurance company (BARMER GEK). All patients hospitalised between 2009 and 2011 with a main diagnosis of PAD of the lower limb or with a main diagnosis of diabetic foot syndrome (DFS) were included. They observed a better cumulative overall survival of patients with PAD (*n* = 24,687) compared to patients with PAD and diabetes (*n* = 8652), with a 4-year survival rate of 70% in patients with PAD compared to 60.8% in patients with PAD and diabetes. Amputation-free survival at 4 years in this study was 86.5% in patients with PAD compared to 74.4% in patients with PAD and DM. However, Malyar et al. [9] did not provide information on the influence of treatment procedures and PAD stage on the results. In our own dataset, 4-year survival rates were 60.1% for DM and 69.6% for non-DM in PAD stage III (*p* < 0.001), but only 39.6% for DM and 48.5% for non-DM in PAD stage IV (*p* < 0.001). The major amputation rates (combined ipsilateral and contralateral amputations) after 4 years for patients were 23.5% vs. 19.8% (DM vs. non-DM) in PAD stage III (*p* = 0.006), and 38.1% vs. 32.1% in stage IV (*p* < 0.001).

This study obviously has several limitations: the completeness of the datasets depends on the coding quality of individual hospitals and the documentation by the health insurance company; coding errors cannot be ruled out. The data reflect the patient clientele of one health insurance company and its social structure and may not necessarily represent the treatment situation for the entire population in Germany. However, AOK is the largest health insurance company in the country, with a market share of 37%. Due to anonymity of the datasets, the treating hospitals and their case volumes could not be analysed. Furthermore, causes of death could not be determined. Regarding the data on amputation, it was not always possible to clearly distinguish between ipsilateral and contralateral amputation in follow-up. Therefore, amputation rates in the follow-up always indicate the combined ipsi- and contralateral amputation rate. Finally, the classification of PAD according to Fontaine, instead of according to Rutherford, was chosen in the present study, as the ICD coding did not allow a distinction between Rutherford 5 and 6.

Nevertheless, this is the largest study on patients with CLTI reporting on perioperative and long-term outcomes of LEB in DM and non-DM patients, considering the bypass level (above-the-knee, below-the-knee, and crural/pedal) and Fontaine stages for PAD classification.

## 5. Conclusions

In summary, significant findings were observed: both perioperative and long-term outcomes in patients with PAD undergoing LEB are consistently worse for DM compared to non-DM. Patients with rest pain have a better outcome than those with gangrene. This applies equally to both DM and non-DM. Furthermore, outcomes are depending on the bypass level: patients with crural/pedal bypasses have poorer survival and higher major amputation rates compared to patients with below- and above-the-knee bypasses, applying to both, DM and non-DM.

In conclusion, the findings of this study help to educate patients with diabetes, help to report the prognosis of bypass surgery to patients before CLTI treatment and underline the influence of diabetes on long-term outcomes. The results of LEB surgery should be compared with those of endovascular surgery.

## Figures and Tables

**Table 1:** Characteristics and comorbidities of CLTI patients with diabetes (DM) or without diabetes (non-DM).

	Non-DM *n* = 15,367	DM *n* = 7266	*p* Value
Male sex, *n* (%)	9930 (64.6)	4593 (63.2)	0.040
Female sex, *n* (%)	5437 (35.4)	2673 (36.8)	0.040
PAD Fontaine stage III, *n* (%)	4641 (30.2)	1419 (19.5)	<0.001
PAD Fontaine stage IV, *n* (%)	10,726 (69.8)	5847 (80.5)	<0.001
Bypass above-the-knee PAD III, *n* (%)	1735 (37.4)	564 (39.7)	0.109
Bypass below-the-knee PAD III, *n* (%)	1525 (32.9)	438 (30.9)	0.160
Bypass crural/pedal PAD III, *n* (%)	1381 (29.8)	417 (29.4)	0.790
Bypass above-the-knee PAD IV, *n* (%)	3292 (30.7)	1655 (28.3)	0.001
Bypass below-the-knee PAD IV, *n* (%)	3232 (30.1)	1634 (27.9)	0.003
Bypass crural/pedal PAD IV, *n* (%)	4202 (39.2)	2558 (43.7)	<0.001
Age, mean ± SD in years; median (min-max)	72.2 ± 10.8; 73 (19–101)	73.2 ± 9.7; 74 (28–100)	<0.001
Age men, mean ± SD in years; median (min-max)	69.6 ± 10.3; 71 (19–99)	71.3 ± 9.4; 73 (28–95)	<0.001
Age women, mean ± SD in years; median (min-max)	76.8 ± 10.3; 78 (27–101)	76.5 ± 9.4; 78 (41–100)	0.015
Arterial hypertension, *n* (%)	6154 (40.0)	6017 (82.8)	<0.001
Left heart failure (NYHA 2-4 and unspecified), *n* (%)	1675 (10.9)	2394 (32.9)	<0.001
History of myocardial infarction, *n* (%)	1058 (6.9)	1487 (20.5)	<0.001
History of stroke, *n* (%)	450 (2.9)	490 (6.7)	<0.001
History of TIA, *n* (%)	217 (1.4)	192 (2.6)	<0.001
COPD, *n* (%)	1542 (10.0)	1250 (17.2)	<0.001
Renal insufficiency (stages 3–5), *n* (%)	1707 (11.1)	2655 (36.5)	<0.001

CLTI critical limb-threatening ischaemia, non-DM patients without diabetes mellitus type 2, DM patients with diabetes mellitus type 2, PAD peripheral artery disease, NYHA New York Heart Association, COPD chronic obstructive pulmonary disease, renal insufficiency (stages 3–5) glomerular filtration rate under 60 mL/min/1.73 m².

**Table 2:** Perioperative outcomes of CLTI patients with diabetes (DM) or without diabetes (non-DM) following LEB.

	Non-DM *n* = 15,367	DM *n* = 7266	*p* Value
Perioperative mortality all patients, *n* (%)	1259 (8.2)	729 (10.0)	<0.001
Perioperative mortality men, *n* (%)	729/9930 (7.3)	418/4593 (9.1)	<0.001
Perioperative mortality women, *n* (%)	530/5437 (9.7)	311/2673 (11.6)	0.009
Perioperative mortality PAD III, *n* (%)	199/4641 (4.3)	76/1419 (5.4)	0.091
Perioperative mortality PAD IV, *n* (%)	1060/10,726 (9.9)	653/5847 (11.2)	0.009
Perioperative mortality above-the-knee bypass, *n* (%)	317/5027 (6.3)	178/2219 (8.0)	0.008
Perioperative mortality below-the-knee bypass, *n* (%)	384/4757 (8.1)	225/2072 (10.9)	<0.001
Perioperative mortality crural/pedal bypass, *n* (%)	558/5583 (10.0)	326/2975 (11.0)	0.163
LOS, mean ± SD in days; median (min-max)	25.6 ± 19.7; 20 (0–298)	28.9 ± 22.2; 22 (1–232)	<0.001
Major amputation, *n* (%)	892 (5.8)	539 (7.4)	<0.001
Minor amputation, *n* (%)	2676 (17.4)	2103 (28.9)	<0.001
Dialysis, *n* (%)	478 (3.1)	536 (7.4)	<0.001
Blood transfusions, *n* (%)	6027 (39.2)	3613 (49.7)	<0.001
Intensive care treatment, *n* (%)	2607 (17.0)	1587 (21.8)	<0.001
Wound infections, *n* (%)	1639 (10.7)	868 (11.9)	0.004
Myocardial infarction, *n* (%)	344 (2.2)	223 (3.1)	<0.001
Complication-free, *n* (%)	10,636 (69.2)	4100 (56.4)	<0.001

CLTI critical limb-threatening ischaemia, non-DM patients without diabetes mellitus type 2, DM patients with diabetes mellitus type 2, PAD peripheral artery disease, LOS length of stay. Complication-free: None of the above analysed complications (except for blood transfusions and intensive care treatment).

**Table 3:** Nine-year survival of CLTI patients with diabetes (DM) or without diabetes (non-DM) following LEB.

	Non-DM *n* = 15,367	DM *n* = 7266	*p* Value
Survival all patients, *n* (%)	6354 (31.1)	2373 (21.5)	<0.001
Survival PAD III, *n* (%)	2652 (45.7)	692 (34.3)	<0.001
Survival PAD IV, *n* (%)	3702 (25.0)	1681 (18.5)	<0.001
Survival above-the-knee bypass PAD III, *n* (%)	1068 (49.4)	290 (37.9)	<0.001
Survival below-the-knee bypass PAD III, *n* (%)	858 (45.5)	211 (33.1)	<0.001
Survival crural/pedal bypass PAD III, *n* (%)	726 (41.3)	191 (32.3)	0.001
Survival above-the-knee bypass PAD IV, *n* (%)	1227 (27.0)	518 (21.5)	<0.001
Survival below-the-knee bypass PAD IV, *n* (%)	1109 (23.4)	474 (17.3)	<0.001
Survival crural/pedal bypass PAD IV, *n* (%)	1366 (24.3)	689 (17.2)	<0.001

CLTI critical limb-threatening ischaemia, non-DM patients without diabetes mellitus type 2, DM patients with diabetes mellitus type 2, PAD peripheral artery disease. Percentages are Kaplan–Meier estimates.

**Table 4:** Multivariate Cox regression analysis of predictors of long-term mortality.

	HR	95% CI	*p* Value
Male gender (vs. female gender)	1.16	1.12–1.21	<0.001
Age (increased by 1 year)	1.04	1.04–1.05	<0.001
Bypass crural/pedal and below-the-knee (vs. above-the-knee)	1.02	0.98–1.06	0.323
PAD Fontaine stage IV (vs. III)	1.62	1.55–1.69	<0.001
Diabetes mellitus type 2	1.10	1.05–1.14	<0.001
Left heart failure (NYHA 2-4 and unspecified)	1.28	1.22–1.35	<0.001
COPD	1.35	1.29–1.42	<0.001
Renal insufficiency (stages 3–5)	1.44	1.38–1.51	<0.001
Arterial hypertension	0.90	0.87–0.94	<0.001
History of myocardial infarction	1.05	0.99–1.11	0.102
History of stroke	1.18	1.09–1.28	<0.001

HR hazard ratio, CI confidence interval, PAD peripheral artery disease, NYHA New York Heart Association, COPD chronic obstructive pulmonary disease, renal insufficiency (stages 3–5) glomerular filtration rate under 60 mL/min/1.73 m².

**Table 5:** Nine-year major amputation rates of CLTI patients with diabetes (DM) or without diabetes (non-DM) following LEB.

	Non-DM *n* = 15,367	DM *n* = 7266	*p* Value
Major amputation rates all patients, *n* (%)	4114 (39.0)	2197 (45.3)	<0.001
Major amputation rates PAD III, *n* (%)	976 (29.9)	319 (31.5)	0.005
Major amputation rates PAD IV, *n* (%)	3138 (43.7)	1878 (49.3)	<0.001
Major amputation rates above-the-knee bypass PAD III, *n* (%)	257 (22.4)	83 (23.5)	0.283
Major amputation rates below-the-knee bypass PAD III, *n* (%)	297 (29.9)	90 (32.7)	0.117
Major amputation rates crural/pedal bypass PAD III, *n* (%)	422 (39.8)	146 (40.7)	0.018
Major amputation rates above-the-knee bypass PAD IV, *n* (%)	715 (35.2)	432 (40.7)	<0.001
Major amputation rates below-the-knee bypass PAD IV, *n* (%)	856 (41.1)	463 (49.6)	0.002
Major amputation rates crural/pedal bypass PAD IV, *n* (%)	1567 (52.4)	983 (55.5)	0.007

CLTI critical limb-threatening ischaemia, non-DM patients without diabetes mellitus type 2, DM patients with diabetes mellitus type 2, PAD peripheral artery disease. Percentages are Kaplan–Meier estimates.

## Data Availability

Restrictions apply to the availability of these data. Data was obtained from WIDO on behalf of AOK.
